# A Novel Method to Monitor the Evolution of Antimicrobial Resistance in *Acinetobacter baumannii* Biofilms

**DOI:** 10.3390/ijms27031512

**Published:** 2026-02-03

**Authors:** Raul Anguita, Jiarui Li, Ester Boix, Guillem Prats-Ejarque

**Affiliations:** Department of Biochemistry and Molecular Biology, Faculty of Biosciences, Universitat Autònoma de Barcelona (UAB), 08193 Cerdanyola del Vallès, Spain; raul.anguita@uab.cat (R.A.); jiarui.li@uab.cat (J.L.)

**Keywords:** biofilms, antimicrobial peptides, RNase, protein chimera, antibiotic resistance, colistin

## Abstract

Biofilms are microbial communities embedded in an extracellular matrix that facilitates their attachment to surfaces. This lifestyle provides advantages to pathogenic bacteria, including increased survival in the presence of antibiotics and an enhanced capacity to develop resistance. Once a biofilm is established, infections get difficult to eradicate and frequently become chronic. There is, therefore, an urgent need to develop novel strategies to counteract biofilm-associated antibiotic resistance. Here, we developed a method to monitor the evolution of antimicrobial resistance, aiming to evaluate novel drugs against bacterial resistance to antibiotics. We validated this methodology using an RNase chimera with antibiofilm activity and a reported ability to hinder colistin resistance in planktonic cultures of *Acinetobacter baumannii* (*A. baumannii*). We assessed the emergence of resistance in *A. baumannii* biofilms by repeated cycles of colistin exposure. This method not only preserves biofilm structure throughout treatment but also enables controlled induction of resistance acquisition while monitoring antimicrobial efficacy. Although the RNase enhanced the antibiotic’s activity against biofilms by reducing by 50% the effective dose, it did not prevent the emergence of colistin resistance, indicating that the protein may use distinct mechanisms against planktonic and biofilm communities. Nonetheless, our findings highlight the potential of this methodology for evaluating antibiotic-adjuvant candidates to combat antibiotic resistance in biofilms.

## 1. Introduction

Bacterial resistance refers to the acquired ability of microorganisms to grow in the presence of antibiotic concentrations that would normally be inhibitory or lethal [1]. Infections caused by multidrug-resistant (MDR) bacteria are among the leading causes of death worldwide and pose a serious threat to healthcare systems [2]. Particularly concerning is the emergence of resistance to last-resort antibiotics [3,4].

Antibiotic resistance can arise through point mutations or the horizontal transfer of resistance genes, activating a variety of mechanisms that impair antibiotic efficacy. These include reduced membrane permeability, efflux pump activation, target modification and enzymatic drug inactivation [1]. Another major strategy used by bacteria to withstand antibiotics is biofilm formation. Biofilms are communities of bacteria that adhere to biotic or abiotic surfaces and are embedded in a self-produced extracellular polymeric matrix (ECM). This structure acts as a physical barrier, significantly reducing antibiotic penetration and rendering biofilm-associated bacteria much less susceptible than their planktonic counterparts [5]. In addition, nutrient deprivation and hypoxic conditions in deeper biofilm layers can induce bacterial dormancy, further increasing antibiotic tolerance [6,7,8,9,10]. The biofilm lifestyle also promotes higher mutation rates and enhances horizontal gene transfer, accelerating the acquisition of resistance and, in some cases, reducing dependence on the ECM itself for protection [11,12,13,14].

Biofilms are common in hospital environments and grow on surfaces such as medical instruments, implantable and prosthetic devices and wastewater systems. They are key contributors to the nosocomial transmission of bacterial infections [15,16,17]. Clinically, biofilms are implicated in a range of persistent infections [18]. According to the U.S. National Institutes of Health (NIH), approximately 80% of bacterial infections in humans are biofilm-related [19]. Given the serious public health threat posed by pathogenic biofilms and the limited efficacy of current antibiotics, there is an urgent need to develop novel therapeutic strategies specifically targeting this bacterial lifestyle.

Among the compounds attracting increasing interest in the search for novel antimicrobial therapies, antimicrobial peptides (AMPs) stand out as some of the most promising candidates. Although AMPs are not universally effective against biofilms, several have been identified that can inhibit biofilm formation or facilitate their removal. Some AMPs even exhibit specific anti-biofilm activity, with minimum biofilm inhibitory concentrations (MBIC) lower than their minimum inhibitory concentrations (MIC) [20].

Some of these anti-biofilm AMPs interfere with QS systems or secondary messenger molecules, thereby disrupting the coordinated signalling required for biofilm development [21,22,23,24]. Enzymes with proteolytic, DNase or polysaccharide-degrading activity are also promising as anti-biofilm agents due to their ability to break down the ECM and restore bacterial susceptibility to antibiotics [25,26]. Other enzymes, such as lactonases, can inhibit QS signalling, thereby preventing biofilm formation or dismantling pre-established ones [27,28]. Importantly, AMPs and antimicrobial enzymes tend to exert less selective pressure for resistance development compared to conventional antibiotics. This is largely due to their broad-spectrum and nonspecific modes of action, targeting microbial membranes, structural polymers or signalling molecules, rather than specific protein targets prone to mutation [29,30]. Given the challenges associated with translating AMPs into clinical use, a promising strategy is their combination with conventional antibiotics to enhance therapeutic efficacy, minimize toxicity and reduce the likelihood of resistance emergence [31].

In this context, the development of a simple, rapid and reproducible method to screen novel compounds, including AMPs, for their ability to hinder antibiotic resistance in biofilms holds significant promise for advancing antimicrobial research. To address this need, we established an in vitro biofilm resistance evolution assay that enables quantitative monitoring of antimicrobial efficacy across multiple exposure cycles, and we validated this approach using engineered RNase chimeras from the human RNase A superfamily. RNase hybrids that merge the catalytic core of RNase 1 with membrane-targeting regions of RNase 3, had previously demonstrated the ability to enhance colistin activity and delay resistance development in *Acinetobacter baumannii* (*A. baumannii*) planktonic cultures [32,33,34]. Among the engineered chimeric RNase 3/1 variants, RNase 3/1-v3 is the most antibacterial protein [34] and was selected for testing the developed protocol. Here, we assessed for the first time their potential to hinder resistance acquisition within biofilms. The assay presented in this work provides a robust and highly reproducible platform for testing biofilm antibiotic resistance acquisition, allowing high-throughput screening with high number of replicas, with no need to directly manipulate the biofilm between cycles.

## 2. Results

### 2.1. Validating a Resazurin-Based Method to Test Biofilm Viability

**Figure 1:**
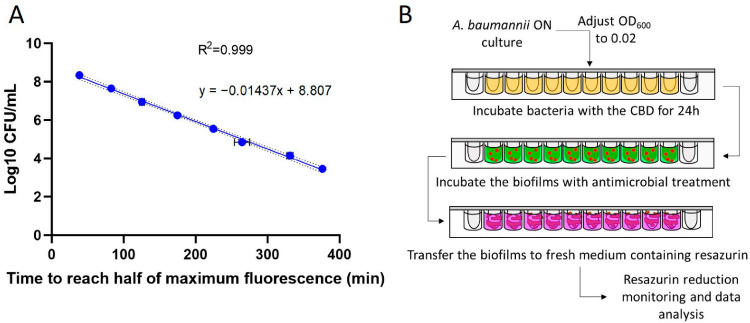
(**A**) **Correlation between planktonic bacteria and the time required to reach half of maximum fluorescence signal.** Linear regression analysis was performed; the equation and R^2^ value are indicated. Error bars represent ± standard error of the mean (SEM) for each data point. Three technical replicates were done. (**B**) **Schematic representation of the resazurin-based assay developed to assess biofilm viability following antimicrobial treatment.** The bacteria were grown overnight in medium (light orange) to develop biofilms in the CBD. After 24 h, these mature biofilms were treated with medium containing antimicrobial agents (green with red dots). Their viability was then assessed by measuring fluorescence in resazurin-containing medium (violet), which indicates the activity of live bacteria within the biofilms.

To evaluate the ability of potential antibiotic-adjuvants to hinder colistin resistance in *A. baumannii* biofilms, we developed a methodology that allows the assessment of biofilm viability while preserving structural integrity and enabling continuous cycles of colistin exposure. To this end, we grew biofilms using the Calgary Biofilm Device (CBD) and monitored viability through a resazurin-based fluorescence assay based on a previously established methodology [35]. To determine bacterial loads within biofilms, we established a correlation line using planktonic cultures. By this way, serial dilutions of bacterial suspensions were prepared and their concentrations determined by colony counting. The time required for each dilution to reach half-maximal fluorescence, used as a proxy for resazurin conversion rate, was monitored via continuous kinetic readings. This approach yielded a near-perfect correlation (R^2^ = 0.999) and the resulting equation was used to estimate the planktonic-equivalent number of bacteria in biofilm samples (Figure 1A). Once this correlation was confirmed, we adapted a previously described protocol [35] to assess the impact of antimicrobials on biofilm viability. In this assay, biofilms treated with antimicrobial agents were sequentially transferred to a plate containing fresh medium and resazurin and fluorescence kinetics were monitored (Figure 1B). The planktonic-equivalent bacterial load was inferred from the time required to reach half-maximal fluorescence. This allowed us to quantify the percentage of biofilm viability relative to untreated controls.

**Figure 2:**
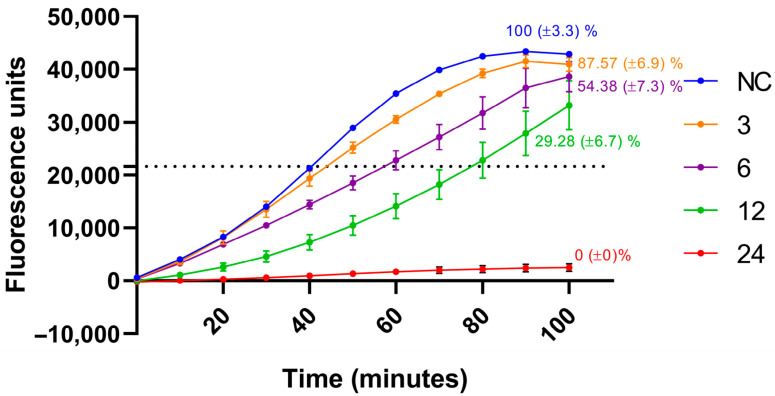
**Assessment of fluorescence signal generated by bacterial biofilms pre-treated with colistin.** Biofilms were exposed to serial dilutions of colistin (ranging from 24 to 3 µM) for 12 h, then transferred to fresh medium containing resazurin to assess viability via fluorescence monitoring. The fluorescence level corresponding to half of the maximum resazurin reduction is indicated by a dotted line. Biofilm viability following each colistin treatment was determined by analysing the fluorescence kinetics, with calculated viability percentages indicated next to each curve. Error bars represent ± standard error of the mean (SEM) for each data point. Three biological replicates were performed. The time required to reach half of the maximum fluorescence signal was extrapolated to bacterial concentration using the calibration curve established in Figure 1A. Final biofilm viability was expressed as the percentage of viable bacteria in treated samples relative to the untreated control.

Next, we tested the capacity of the method to quantify the effect of antimicrobial treatments against biofilms. *A. baumannii* biofilms were exposed to a range of colistin concentrations. As shown in Figure 2, biofilm viability decreased progressively with increasing colistin concentrations. No fluorescence signal was detected following treatment with 24 µM colistin, which was therefore designated as the minimum biofilm eradication concentration (MBEC) for this antibiotic.

**Figure 3:**
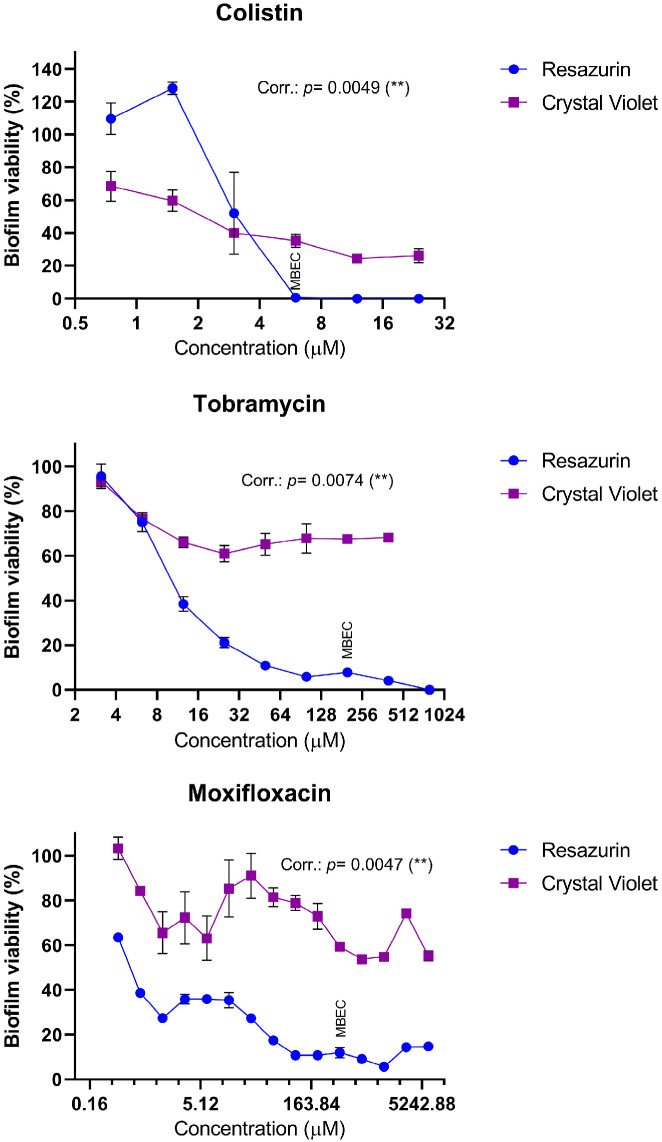
**Biofilm viability and biomass after antibiotic treatment, assessed using resazurin-based and crystal violet-based assays.** Biofilms were exposed to serial dilutions of colistin, tobramycin or moxifloxacin in MHB for 12 h. After treatment, biofilms were either transferred to fresh MHB containing resazurin for fluorescence monitoring or subjected to CV staining and distaining. The correlation (Corr.) between both methods for evaluating biofilm viability was analysed using the Pearson correlation test, with significance values (*p*) indicated on each graph. ** indicates statistical significance. Error bars represent ± SEM. Three technical replicates were done for each experiment. MBEC values for each antibiotic, determined by colony counting method, are also indicated.

We then validated the resazurin-based assay by comparing it with a well-established method for assessing biofilm biomass: crystal violet (CV) staining. *A. baumannii* biofilms were exposed to a range of concentrations of colistin, tobramycin and moxifloxacin and then analysed using both methods to quantify biofilm mass and viability. The results from the resazurin assay correlated well with those from the CV assay, as both techniques showed significant agreement in measuring biofilm status following exposure to colistin, tobramycin and moxifloxacin (Figure 3). MBECs, determined by the reference method based on biofilm detachment and colony counting, were 6 µM (7.62 µg/mL) for colistin, 200 µM (93.5 µg/mL) for tobramycin and 400 µM (160.63 µg/mL) for moxifloxacin. At these concentrations, the resazurin assay detected nearly complete loss of biofilm viability, whereas CV staining still indicated substantial biofilm biomass. Based on these findings, we conclude that the resazurin-based assay is a reliable method for assessing biofilm viability.

Considering that colistin presented the lowest MBEC values of the tested antibiotics, we selected it for further combinatorial assays and evaluation of the biofilm ability to acquire resistance in an in vitro exposure evolution assay.

### 2.2. Antibiofilm Activity of Human RNases

To validate our methodology, we first tested the efficacy of an antimicrobial protein chimera under study in our laboratory. In our prior work, we designed three RNase 3/1 chimera, from which version 1 and 3 variants were demonstrated to enhance colistin activity and delay resistance development in planktonic cultures [32,33,34,36]. In this study, we wanted to test a resazurin-based method for assessing bacterial resistance in biofilms. For this reason, we used our RNase chimera, together with their parental proteins, to validate the method by testing its biofilm-targeting capacity and ability to delay the biofilm resistance acquisition.

**Figure 4:**
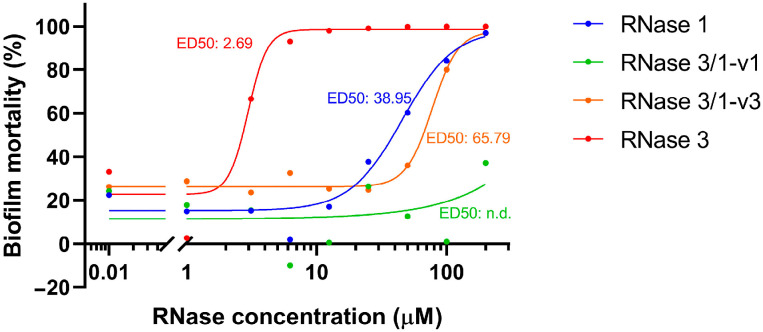
**Dose-dependent effect of recombinant RNases on bacterial biofilms**. Biofilms were exposed to varying concentrations of RNases (0.01 to 200 µM) in HBS for 4 h. Following treatment, biofilms were transferred to fresh MHB containing resazurin for viability assessment via fluorescence monitoring. The ED_50_ values were calculated using a non-linear regression model based on normalized response data.

Before proceeding to the resistance assay, we needed to assess the biocidal activity of the parental and chimeric RNases against *A. baumannii* biofilms using the resazurin-based assay. RNase 3 exhibited the strongest anti-biofilm effect, with an ED_50_ of 2.69 µM, followed by RNase 1 (ED_50_: 38.95 µM) and RNase 3/1-v3 (ED_50_: 65.79 µM) (Figure 4). The notable activity of RNase 1 was surprising, given that it lacks activity against bacterial suspensions, unlike the other RNases tested [32]. In contrast, RNase 3/1-v1 demonstrated minimal biofilm-killing capacity, with no detectable ED_50_.

### 2.3. Combinatorial RNase-Colistin Therapy Against Biofilms

To assess whether RNases can enhance colistin’s ability to eradicate *A. baumannii* biofilms, various concentrations of colistin were combined with different concentrations of chimeric or parental RNases. Among the tested proteins, RNase 1 and RNase 3/1-v3 showed the clearest adjuvant effect, enhancing biofilm killing and reducing the colistin MBEC. RNase 1 also displayed a dose-dependent potentiation of colistin activity, whereas the effects of RNase 3/1-v1 and RNase 3 were less pronounced and did not show a significant RNase dose dependency (Figure S1). Based on these results, further combinatorial experiments with the RNase chimeras were conducted using a fixed concentration of 1 µM.

**Figure 5:**
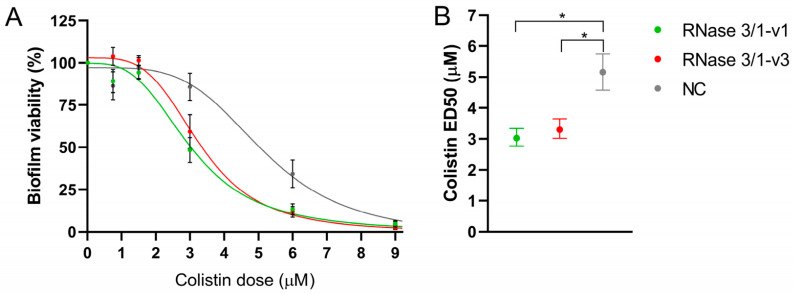
**Contribution of RNase 3/1-v1 and RNase 3/1-v3 to colistin-mediated biofilm eradication.** Biofilm viability profiles (**A**) and the corresponding ED_50_ values (**B**) for colistin in the presence or absence of each RNase are shown. Biofilms were treated with a fixed RNase concentration of 1 µM and varying concentrations of colistin. Data were analysed using non-linear regression and are presented as mean ± SEM. ED_50_ values were derived from the fitted curves, with error bars indicating the 95% confidence interval (CI). Statistical significance is indicated by an asterisk (*) when 95% CIs do not overlap. Five biological replicates were done, with three technical replicates for each assay.

None of the RNases improved colistin’s MBEC (Figure 5A). However, supplementation with RNase 3/1-v1 and RNase 3/1-v3 reduced the colistin concentration needed to achieve 50% biofilm killing (ED_50_) by 1.7-fold and 1.6-fold, respectively (Figure 5B). Besides, no significant differences were observed between the RNase 3/1-v1 and RNase 3/1-v3.

### 2.4. Evaluation of the Biofilm Resistance Evolution Assay Using Colistin/RNase Combined Treatment

**Figure 6:**
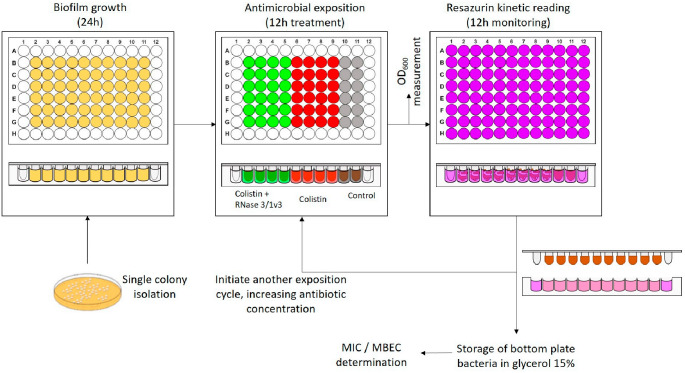
**Schematic representation of the biofilm resistance evolution assay under colistin exposure.** Bacteria from a single colony isolate were grown to the biofilm state in MHB (light orange) using the Calgary Biofilm Device (CBD). Biofilms were exposed to either colistin alone (red) or colistin supplemented with 1 µM RNase 3/1-v3 (green). Untreated controls were exposed to MHB without antimicrobials (grey). After 12 h of treatment, the biofilms were transferred to fresh MHB containing resazurin for an additional 12 h incubation to assess viability via fluorescence monitoring (light violet indicates absence of biofilms in blank controls or non-viable biofilms and pink indicates presence of viable biofilms). Subsequently, biofilms were moved to a new 96-well plate with fresh antimicrobial treatment. The colistin concentration was increased if no reduction in bacterial viability was observed or maintained if a substantial decrease was detected. Planktonic bacteria present in the bottom plates were quantified by OD_600_ measurements and stored in 15% glycerol at −80 °C for subsequent MIC determination.

To evaluate the Biofilm Resistance Evolution assay we applied repeated biofilm exposure to colistin for 9 days, using RNase 3/1 as a model adjuvant candidate (Figure 6). We selected RNase 3/1-v3 for its superior antimicrobial activity in both planktonic cultures [34] and biofilms (Figure 4). Pre-formed biofilms in the CBD were treated with colistin for 12 h, with or without RNase 3/1-v3. Viability was then assessed over 12 h using resazurin-based fluorescence monitoring in a Spark^®^ microplate reader. Recovered biofilms were transferred to fresh colistin treatments, adjusting the antibiotic concentration based on the previous cycle’s efficacy. This process was repeated for nine cycles. After each cycle, bacteria were preserved in glycerol at −80 °C for later MIC or MBEC testing.

Only limited biofilm mortality was observed in response to increasing colistin concentrations throughout the resistance evolution assay, with no significant differences between treatment groups (Figure S2). Four out of the 24 total initial biofilm replicates (83.33%) remained viable after the first antibiotic exposure in both the colistin-only and the RNase 3/1-v3-supplemented groups. No additional mortality was recorded until the final two days of the assay. By the end of the experiment, 66.66% of the colistin-only replicates and 79.29% of the RNase 3/1-v3-supplemented replicates remained viable.

**Figure 7:**
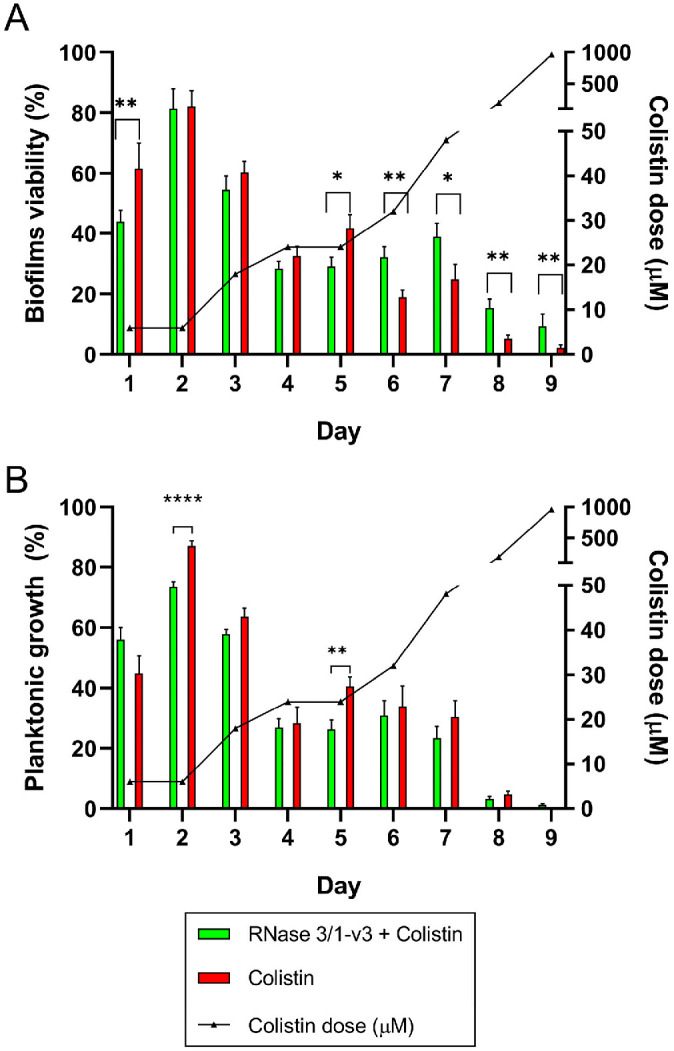
**Effect of repeated colistin exposure on *Acinetobacter baumannii* (*A. baumannii*) biofilms in the presence or absence of RNase 3/1-v3.** (**A**) **Biofilm viability after each day of colistin treatment.** (**B**) **Planktonic bacterial growth measured by OD_600_ after each exposure cycle.** The corresponding colistin concentration used at each day is indicated by triangles on the right axis. Error bars represent the ±SEM. Statistical significance was assessed using the non-parametric Mann–Whitney U test for comparisons between treatments on the same day (**** *p* < 0.0001, ** *p* < 0.01, * *p* < 0.05, ns *p* > 0.05). 24 biological replicates were done for each treatment, and 12 biological replicates for the negative control.

A reduction in biofilm viability due to the presence of RNase 3/1-v3 was observed during the first days of the assay (days 1 to 5), with significant differences compared to biofilms exposed to colistin alone occurring on days 1 and 5 (Figure 7A and Figure S3). During this period, colistin concentrations were increased from 6 to 24 µM. However, this trend reversed in the latter half of the assay (days 6 to 9), where RNase 3/1-v3 supplementation not only failed to enhance colistin’s effect on biofilms but actually resulted in a significant increase in biofilm viability. These later days corresponded to higher colistin doses, ranging from 32 to 960 µM. RNase 3/1-v3 supplementation also affected planktonic bacteria derived from the biofilms, causing an overall reduction in bacterial growth (measured by OD_600_), which was significant on days 2 and 5 of the assay (Figure 7B). On days 8 and 9, when colistin concentrations were 192 and 960 µM, respectively, planktonic bacterial growth was nearly undetectable.

**Figure 8:**
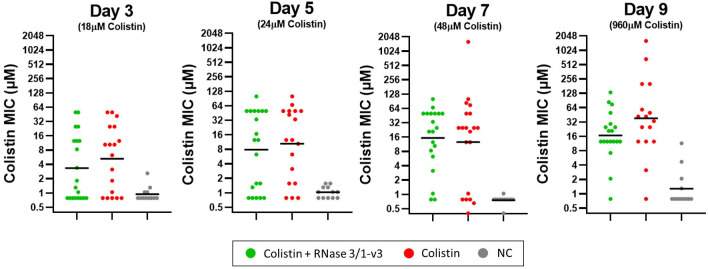
**Colistin MICs of *A. baumannii* strains collected on days 3, 5, 7 and 9 of the biofilm resistance evolution assays.** The geometric mean MIC is indicated for each group. Statistical comparisons between the colistin-only and colistin + RNase 3/1-v3 treatments were performed using the non-parametric Mann–Whitney U test, yielding the following *p*-values: 0.4543 (Day 3), 0.6675 (Day 5), 0.7824 (Day 7) and 0.1062 (Day 9). MIC values of each biological replicate correspond to the average of three technical replicates.

Analysis of colistin MICs in bacterial isolates collected on days 3, 5, 7 and 9 of the resistance evolution assay revealed no statistically significant differences in colistin resistance between lineages exposed solely to the antibiotic and those also treated with RNase 3/1-v3 (Figure 8). However, the lineages that developed the highest levels of resistance, able to grow at colistin concentrations above 128 µM, were exclusively from the group not exposed to the RNase.

**Figure 9:**
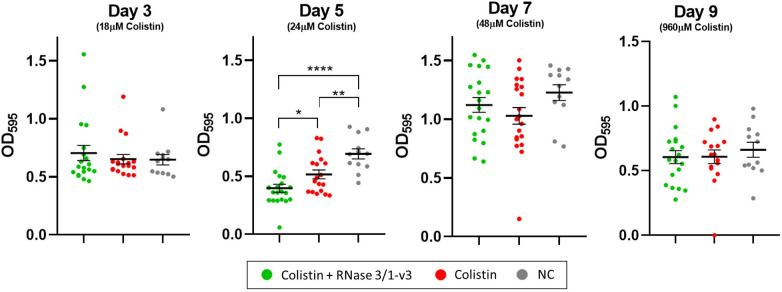
**Biofilm-forming capacity of *A. baumannii* strains collected from the biofilm resistance evolution assay on days 3, 5, 7 and 9.** Biofilm biomass of each biological replicate was quantified using the CV assay. Mean values and ±SEM are shown. Statistical significance between treatment groups for each time point was assessed using the Mann–Whitney U test (**** *p* < 0.0001, ** *p* < 0.01, * *p* < 0.05, ns *p* > 0.05).

We then assessed whether RNase 3/1-v3 supplementation affected the bacteria’s ability to form biofilms. Bacterial lineages derived from biofilms exposed to both colistin and RNase 3/1-v3 during the 5th day of the resistance evolution assay showed a reduced capacity to form biofilms compared to those exposed only to the antibiotic or the control (Figure 9). Lineages from day 5 exposed solely to colistin also exhibited a diminished ability to form biofilms relative to the control. No significant differences were observed between control and treated biofilms for the rest of the days of the resistance assay. Interestingly, diminished biofilm formation capacity correlated with higher colistin MIC values (Figure S4), which is in agreement with other studies [37,38].

The results suggest that day 5 of the resistance evolution assay marked the most significant impact of RNase 3/1-v3 supplementation in limiting biofilm development and the acquisition of colistin resistance. This conclusion is supported by differences observed in bacterial viability during treatment, both in planktonic and biofilm states (Figure 7), as well as in the biofilm-forming capacity of the evolved strains (Figure 9).

**Figure 10:**
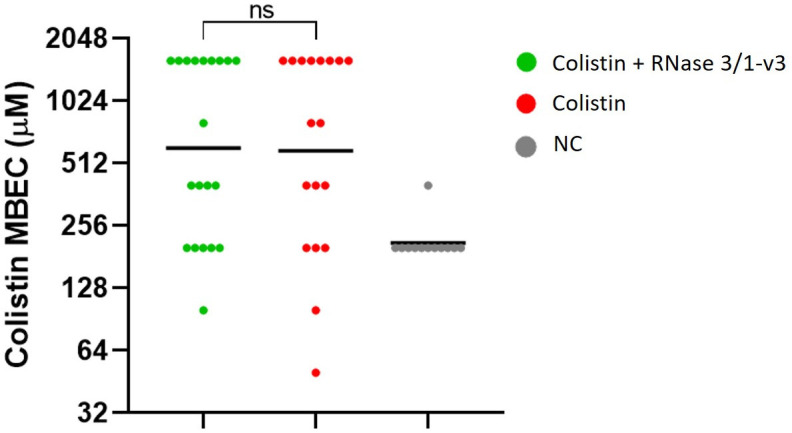
**Colistin MBEC values of *A. baumannii* biofilms derived from each biological replicate of Day 5 strains from the biofilm resistance evolution assay.** Geometric means are indicated. Statistical analysis was performed using the Mann–Whitney U test. (ns *p* > 0.05).

To further investigate potential effects on biofilm susceptibility, colistin MBECs were determined for these strains. Biofilms were regrown from glycerol-stored cultures and exposed to a range of colistin concentrations (1.56 to 1597.4 µM). However, no differences in MBEC values were observed between bacteria exposed to colistin alone and those treated in combination with RNase 3/1-v3 (Figure 10). Notably, control lineages exhibited unexpectedly high MBECs compared to their original values (12–24 µM), though still lower than those of the colistin-treated strains.

These findings suggest that RNase 3/1-v3 supplementation did not significantly influence the acquisition of biofilm-associated colistin resistance. Notably, a positive correlation between MIC and MBEC values was observed for the bacterial isolates collected on day 5 of the resistance assay, indicating that colistin resistance mechanisms developed under biofilm conditions may act in concert with those emerging in planktonic cells (Figure S5).

## 3. Discussion

Biofilm-based infections pose a major public health challenge. Pathogenic biofilms contribute significantly to the spread of infections in hospital settings due to their ability to colonize a wide range of surfaces, including medical devices and their resilience against disinfectants [5,15,16,17]. Once established, these infections are often persistent or even become chronic, largely because biofilms are inherently resistant to antibiotics and immune responses. This resistance is primarily due to the protective nature of the extracellular matrix (ECM) [5,18,39]. Moreover, biofilms serve as hotspots for the development of antimicrobial resistance. They can induce hypermutator phenotypes [10], increase antibiotic tolerance [6,7,8,9,10] and facilitate the transfer of resistance genes [11,12,13]. In this context, developing compounds that can disrupt biofilm organization represents a promising strategy in the fight against pathogenic bacteria and antimicrobial resistance, ultimately improving patient outcomes and reducing healthcare costs [40].

Among the most promising alternatives to traditional antibiotic therapies are antimicrobial proteins and peptides (AMPs), including antimicrobial enzymes. These compounds can disrupt biofilm structures by targeting various mechanisms, such as interfering with signalling molecules essential for biofilm formation and degrading components of the ECM [41]. One particularly promising clinical strategy is the co-administration of AMPs with conventional antibiotics [31]. This combined therapy may enhance antibiotic efficacy and reduce the emergence of resistant strains by promoting biofilm dispersion.

To impulse the identification of novel adjuvants with potential to counter biofilm-related antibiotic resistance, we aimed to optimize a method for rapidly and reproducibly assessing biofilm viability along multiple rounds of antibiotic exposure. Among the commonly used techniques for biofilm quantification, a resazurin-based assay was selected as the most suitable. Resazurin is a non-toxic compound that allows for easy assessment of bacterial viability by measuring the fluorescent signal generated from its reduction to resorufin [35,42]. To facilitate the sequential transfer of treated bacterial cultures across assay cycles, the Calgary Biofilm Device (CBD) was chosen as the platform for biofilm growth [43].

To quantify the number of viable bacteria within biofilms after antimicrobial treatment, some studies have proposed establishing a correlation between bacterial viability and the time required to reach half-maximal resazurin reduction [42,44].

We initially sought to establish this correlation directly using biofilm samples, as reported by other authors [42]; however, we encountered substantial technical limitations. In particular, we were unable to reproducibly generate biofilms spanning a sufficiently broad and well-defined range of bacterial densities. Besides, a reliable CFU quantification proved challenging due to the high variability and limited reliability of biofilm detachment protocols. Given these constraints, we adopted an alternative approach proposed by other researchers: deriving the correlation from planktonic-equivalent bacterial cultures [45]. This method yielded a near-perfect linear regression, resulting in an equation that accurately translates resazurin fluorescence into the number of planktonic-equivalent bacteria within biofilms (Figure 1A).

Next, we developed a protocol based on biofilms grown in Calgary Biofilm Devices (CBD) and exposed for repeated cycles to antimicrobial treatments. After treatment, the biofilms were transferred into fresh MHB containing resazurin to monitor fluorescence (Figure 1B). Finally, the time required to reach half-maximal fluorescence was extrapolated into biofilm viability using the previously established correlation (Figure 1A). To avoid potential bias arising from subtle differences in resazurin reduction rates between planktonic and biofilm lifestyles, as reported by other researchers [41], bacterial counts were normalized relative to non-treated control biofilms. This normalization mitigates potential limitations associated with presenting the data as absolute quantifications. To validate the method, biofilms were treated with a range of colistin concentrations and then monitored for resazurin reduction. As expected, higher colistin concentrations resulted in a greater delay in the fluorescence signal curve, which corresponded to reduced biofilm viability (Figure 2).

To further evaluate the reliability of this approach, we compared the resazurin-based method with the crystal violet (CV) staining assay; this technique has been widely used for decades to quantify biofilm biomass. Biofilms were exposed to a concentration gradient of three different antibiotics: colistin, tobramycin and moxifloxacin. Both methods successfully detected the impact of increasing antibiotic concentrations on biofilms (Figure 3). The CV assay measured changes in total biomass, while the resazurin assay directly assessed bacterial viability. Notably, the results obtained from both techniques showed a positive correlation. However, the resazurin-based method proved more effective in detecting complete biofilm eradication. Minimal or no fluorescence was observed at antibiotic concentrations where biofilms were fully eradicated, as confirmed by colony counting. In contrast, the CV assay still led to strong OD_595_ absorbance signal even at concentrations above the Minimum Biofilm Eradication Concentration (MBEC). This discrepancy likely reflects the ability of CV staining to detect remaining adherent biomass, regardless of bacterial viability [46]. Based on these findings, we concluded that the resazurin-based assay was suitable for our assay.

In our laboratory, we have developed RNase chimeras capable of interfering with *A. baumannii*’s adaptive response to colistin exposure, resulting in a significant reduction in the bacteria’s ability to develop resistance to the antibiotic [32,36].These chimeras were engineered by combining structural elements from human RNase 1 and RNase 3, yielding proteins with both potent catalytic activity and membrane-disruptive properties [33,34]. The initial construct, RNase 3/1-v1, was later optimized through structure-based redesign, leading to the improved RNase 3/1-v3 variant [32,34]. In the present study, these well-characterized RNase chimeras were employed as a functional tool to validate our biofilm-based resistance evolution assay. Given their previously demonstrated capacity to modulate colistin resistance in planktonic bacteria, we reasoned that they would serve as a functional tool to assess the assay utility in monitoring colistin efficacy and resistance dynamics within biofilms.

Next, *A. baumannii* biofilms were exposed to recombinant RNase 1, RNase 3 and the chimeras RNase 3/1-v1 and RNase 3/1-v3 to evaluate their intrinsic bactericidal activity against biofilms. RNase 3 exhibited the strongest activity, nearly achieving complete biofilm eradication at low micromolar concentrations (Figure 4). These results are consistent with previous studies reporting potent bactericidal and anti-biofilm properties for RNase 3, attributed to its high cationicity, amphipathicity and ability to bind to lipopolysaccharides and agglutinate Gram-negative bacteria [47,48]. Surprisingly, RNase 1 demonstrated a greater ability to disrupt biofilms than both RNase 3/1 chimeras, achieving 50% biofilm killing at 38.95 µM. In contrast, RNase 3/1-v3 required 65.79 µM to reach the same effect and RNase 3/1-v1 showed no detectable activity. These findings were unexpected, as both chimeras are known to strongly disrupt bacterial membranes, while RNase 1 has been reported to have minimal antimicrobial properties [49].

Although RNase 3/1 chimera did not outperform the parental RNases in anti-biofilm activity, these results underscore the potential of the RNase A scaffold for developing novel anti-biofilm proteins. Altogether, these findings highlight the importance of further characterizing the specific structural features responsible for the anti-biofilm activities of RNase 3 and RNase 1. Such insights could guide the design of next-generation RNase 3/1 chimeras with enhanced efficacy that surpasses that of the parental proteins. Despite the limited bactericidal activity exhibited by RNase 3/1-v1 and RNase 3/1-v3 against biofilms, we proceeded with further experiments using these chimeric constructs due to their previously demonstrated ability to hinder colistin resistance in *A. baumannii* [32]. Preliminary combinatorial assays suggested that RNase 3/1-v3 had a greater capacity than RNase 3/1-v1 to potentiate colistin activity against biofilms in a dose-dependent manner, with an apparent reduction in colistin MBEC, particularly at RNase concentrations above 1.6 µM (Figure S1). In contrast, subsequent combinatorial assays performed at a fixed RNase concentration of 1 µM showed that both chimeras comparably enhanced colistin-mediated killing, reducing its ED_50_ by 1.7-fold and 1.6-fold for RNase 3/1-v1 and RNase 3/1-v3, respectively (Figure 5), without affecting the MBEC against *A. baumannii* biofilms. ED_50_ and MBEC represent complementary but non-equivalent measures of antibiotic activity against biofilms: while a reduction in ED_50_ reflects increased killing at lower antibiotic concentrations, it does not necessarily translate into complete biofilm eradication. This distinction may explain why, at 1 µM, RNase 3/1 chimeras enhanced colistin efficacy without reducing the MBEC, as highly tolerant biofilm subpopulations are likely to define the eradication threshold.

This outcome contrasts with previous observations in planktonic cultures, where the RNase 3/1 chimeras markedly potentiated colistin activity and substantially reduced its MIC [32] at even lower RNase concentrations. The more modest enhancement observed in biofilms may therefore reflect limited RNase penetration due to the extracellular matrix and the inherently higher resilience of biofilm-associated cells. Notably, despite differences in bactericidal and catalytic activities, RNase 3/1-v1 being more catalytically active and RNase 3/1-v3 more bactericidal [34], both chimeras produced similar potentiation, suggesting that shared structural features primarily underlie this effect. To assess the ability of RNase 3/1-v3 to interfere with biofilm-associated resistance in *A. baumannii*, we developed a biofilm resistance evolution assay (Figure 6). In this assay, pre-formed biofilms were challenged either with colistin alone or with colistin in combination with 1 µM RNase 3/1-v3 over nine exposure cycles. Each cycle consisted of 12 h of antimicrobial treatment followed by 12 h of monitoring by resazurin reduction to assess biofilm viability. To increase selective pressure, the colistin dose was either maintained, if a significant reduction in biofilm viability was observed, or increased, when no substantial changes in biofilm integrity were detected.

This method allows for the simple and rapid passaging of biofilms through progressively increasing antibiotic concentrations. Using a resazurin-based assay provides direct measurements of bacterial viability without requiring biofilm disruption or detachment. As a result, resistance evolution can be monitored in situ during the antibiotic exposure cycles, or ex situ by regrowing biofilms from stored, evolved bacterial populations.

The results showed no cumulative increase in mortality across the treatment cycles for biofilms exposed to colistin plus RNase 3/1-v3 compared to those treated with colistin alone (Figure S2). During the first days of the assay, the presence of RNase 3/1-v3 caused a delay in the resazurin fluorescence kinetics (from days 1 to 5; Figure S3), which, when translated into biofilm viability percentages, resulted in statistically significant reductions compared to biofilms treated with colistin alone on days 1 and 5 (Figure 7A).

These results are consistent with those observed when evaluating the combined RNase-colistin action on biofilms (Figure 5), where RNase chimeras enhanced the initial reduction in biofilm viability in response to colistin but failed to lower the Minimum Biofilm Eradication Concentration (MBEC). Notably, this trend reversed in the later stages of the assay. At higher colistin concentrations, RNase 3/1-v3 not only failed to enhance biofilm killing but appeared to reduce colistin’s activity (Figure 7A). In contrast, analysis of bacterial growth in suspension revealed that RNase 3/1-v3 consistently promoted the killing of planktonic *A. baumannii* cells derived from biofilms throughout the assay period (Figure 7B). These findings align with previous observations in planktonic cultures, where RNase chimeras enhanced bacterial killing in the presence of colistin [32]. The data reinforce the concept that planktonic and biofilm-associated bacterial lifestyles require distinct therapeutic approaches, and several hypotheses may account for the differential efficacy of RNase 3/1-v3 against biofilm-associated versus planktonic cells. The ECM could exert a protective role by restricting the penetration, sequestering or electrostatically repulsing the RNase, thereby reducing its effective availability at the bacterial surface. Besides, the biofilm lifestyle may allow the formation of populations with dormant and stress-response phenotypes more resilient to the antimicrobial therapy. In contrast, under planktonic conditions, the absence of the ECM, increased accessibility of bacterial cells, and more homogeneous metabolic states, could facilitate the interaction of RNase 3/1-v3 with bacterial targets, enhancing the action of colistin.

The biphasic effect observed for RNase 3/1-v3, enhancing colistin-mediated biofilm killing during the early stages of treatment but interfering with its efficacy at later stages, appears to be specific to the biofilm context. Several interconnected hypotheses could explain this phenomenon. First, increasing colistin concentrations could trigger stress-induced protective responses in *A. baumannii* biofilms, such as increased ECM production and activation of resistance pathways, thereby reducing drug penetration and bacterial susceptibility [50,51,52]. Second, as both colistin and RNase 3/1-v3 likely act on similar biofilm-associated targets, high colistin levels may saturate key binding sites, limiting the RNase’s activity. Notably, since this interference is not observed in planktonic bacteria, the saturated targets would be more likely associated with the ECM rather than the bacterial wall. Additionally, a steric hindrance caused by the dense and complex structure of the extracellular matrix may also impair the ability of RNase 3/1-v3 to access and interact effectively with bacteria embedded within the biofilm. Finally, prolonged exposure to colistin and RNase 3/1-v3 may lead to the selection of more tolerant or resistant subpopulations within the biofilm, which could be less responsive to the combined treatment [53]. Altogether, these factors may account for the initial enhancement and subsequent reduction in colistin efficacy observed during the resistance evolution assay. Future experiments should help clarify the underlying mechanisms, such as (i) evaluation of ECM components, exopolysaccharides or extracellular DNA; (ii) assessment of protein penetration into biofilms; (iii) colistin/RNase membrane binding affinity or (iv) monitoring of the emergence of tolerant subpopulations.

Analysis of bacterial strains recovered from the resistance evolution assay revealed no significant differences in the MICs of *A. baumannii* strains whose biofilms were exposed to colistin alone versus those treated with colistin in the presence of RNase 3/1-v3 (Figure 8). However, it is noteworthy that the most resistant phenotypes observed on day 9 of the assay, with colistin MIC values above 128 µM, emerged exclusively in the group exposed to colistin alone. This finding is consistent with previous results obtained using planktonic bacterial cultures [32]. Thus, although the biofilm lifestyle may limit the capacity of RNase 3/1-v3 to hinder resistance development, the absence of highly resistant isolates in the combination treatment suggests that the chimera retains some inhibitory effect, consistent with its behaviour in planktonic populations. We hypothesize that the existence of these colistin-only group resistant isolates are probably due to the total loss of bacterial cell wall, as reported previously [54]. Although the total loss of the bacterial cell wall could make the bacteria totally resistant to colistin, it might also enhance susceptibility to other antimicrobial agents, such as RNase 3/1-v3.

In addition, the bacterial lineages from the resistance evolution assay were also evaluated for their ability to form mature biofilms. CV staining assay revealed that, by day 5, bacteria previously exposed to RNase 3/1-v3 exhibited a reduced capacity for biofilm formation compared to those treated with colistin alone (Figure 9). This impairment in biofilm development may help explain the significant reduction in biofilm viability observed on day 5 of the resistance evolution assay in the RNase-treated group relative to the colistin-only group.

However, despite the differences observed in viability and biofilm development for strains on the 5th day of the assay, no significant differences in colistin MBECs were detected between strains treated with colistin alone and those exposed to the RNase-colistin combination (Figure 10). This suggests that, although RNase 3/1-v3 may transiently impair biofilm establishment and reduce bacterial viability during early treatment phases, it does not appear to prevent the eventual development of colistin resistance within mature biofilms. This reflects a temporary interference with bacterial adaptive responses under combined antimicrobial pressure. At later stages, the intrinsic plasticity of the biofilm lifestyle likely enables adaptation to the treatment, restoring biofilm-forming capacity.

The observation that negative controls exhibited elevated colistin MBEC values (averaging 200 µM), compared to the original range for this *A. baumannii* strain (12–24 µM), suggests that the biofilm resistance evolution assay itself may introduce selective pressure. This increase is possibly due to the repeated enrichment of biofilm-associated cells across treatment cycles, as reported by Yoshida and colleagues [55] in *E. coli* biofilms. The authors observed an increased biofilm forming capacity of selected strains after passaging in a biofilm microfermenter, similarly to what we observe in our negative control.

Although this could seem a putative bias intrinsic to in vitro approaches, similar behaviour has been observed in vivo, as reported by Gloag and colleagues in *Pseudomonas aeruginosa* biofilms in a porcine burn wound model of chronic infection [56].

Nevertheless, strains exposed to colistin developed even higher MBECs, often reaching up to 1597 µM, indicating that the method reliably captures the impact of antibiotic pressure and enables meaningful comparisons between treatment conditions. Moving forward, we aim to further investigate this phenomenon to better understand the selective forces at play during the assay, assess potential effects or unintended consequences and continue validating the robustness of this experimental approach.

The biofilm lifestyle poses a unique therapeutic challenge, partly due to the ECM, which likely limits RNase 3/1-v3 access to embedded bacterial cells. Beyond serving as a physical barrier, ECM components, such as extracellular DNA and other negatively charged polymeric substances, may sequester the cationic RNase through electrostatic interactions, further reducing its availability at the bacterial surface [41,57,58]. Together, these factors highlight the need to optimize the structural features of RNase 3/1-v3 by drawing on the potent biofilm-disruptive properties of its parental enzymes. A deeper understanding of the molecular and structural determinants underlying these activities could inform the design of more effective RNase-based adjuvants. It should also be taken into account that the acquisition of colistin resistance mutations can have a negative impact in the capacity of the bacteria to establish robust biofilms [37]. This is supported by the correlation observed in Figure S4, which evidenced that those bacterial lineages that acquired higher resistance to colistin in planktonic state produced biofilms with reduced biomass. Colistin resistance commonly arise from the activation of LPS modification systems such as PmrCAB or EptA, which promote the addition of positively charged moieties to lipid A [59,60]. Acquisition of plasmid-encoded *mcr* genes also promotes lipid A modification in clinical isolates [61]. In addition, loss-of-function mutations in genes involved in LPS biosynthesis (*lpxA*, *lpxC*, *lpxD*) can lead to complete LPS deficiency [54]. A plausible explanation for the negative impact of these alterations on biofilm formation is that the resulting reduction in the overall negative charge of *A. baumannii* bacterial surface [62] may diminish the bacterial capacity to interact with surfaces to initiate biofilm formation [63]. However, the fact that MBEC and MIC values demonstrated a positive and strong correlation, indicates that biofilm-related and planktonic-related colistin resistance mechanisms apparently collaborate to promote bacterial survival during exposition to high doses of the antibiotic (Figure S5). It is well known that higher antibiotic concentrations are usually required for biofilm eradication compared to bacteria in suspension [64], while subinhibitory colistin doses induce biofilm formation [52]. The relationship between colistin resistance and biofilm formation is complex and still little understood [37]. Further analysis of bacterial resistome could shed light on how each bacterial lifestyle contributes to the emergence of colistin resistance. Future experiments on these evolved strains will focus on the genotypic characterization and its envelope composition. This would help us to understand the underlying resistance mechanisms.

The biofilm resistance evolution assay developed in this study allowed us to effectively evaluate the potential of an antibiotic adjuvant, specifically RNase 3/1-v3, to inhibit the development of colistin resistance within biofilms. An alternative approach used in other studies involves dispersing the biofilm after each treatment cycle and regrowing it from the resulting planktonic population [65]. While this approach is also valid, it may tend to select for traits that enhance biofilm initiation and early-stage development, rather than those that support long-term survival under antibiotic pressure. In contrast, our method more closely mimics the conditions of persistent infections, where biofilms are not entirely eliminated between treatment cycles. It preserves the stable biofilm community structure, including the extracellular matrix, spatial heterogeneity and stress gradients, factors that are critical for biofilm resilience and resistance evolution. This approach also supports the maintenance of slow-growing or dormant persister cells, which may play a key role in the emergence of resistance during prolonged exposure. Additionally, the CBD provides a highly practical platform for conducting biofilm evolution assays. It allows for straightforward implementation of repeated treatment exposures [43]. Other models, such as those using biofilm growth on glass beads [45,66], may be more labour-intensive and less scalable when evolving multiple lineages or screening a broad range of compounds simultaneously.

Overall, while our findings support the potential of AMPs as therapeutic enhancers, they also underscore the distinct challenges posed by biofilm-associated resistance, emphasizing the need to tailor strategies specifically for this bacterial lifestyle. Innovative methodologies, such as the biofilm resistance evolution assay developed in this study, offer a robust platform for evaluating the ability of potential antibiotic adjuvants to hinder the development of antibiotic resistance within biofilms.

## 4. Materials and Methods

### 4.1. Materials

The *A. baumannii* strain (CECT 452; ATCC 15308 was obtained from the Spanish Type Culture Collection (CECT). The *Escherichia coli* BL21 (DE3) strain and the pET11c plasmid were purchased from Novagen. The gene encoding RNase 3/1-v1 was acquired from NZYTech (Lisboa, Portugal) and served as the template for generating the RNase3/1-v3 gene by genetic engineering [34]. The human RNase 1 gene was kindly provided by Dr. Maria Vilanova (Universitat de Girona, Girona, Spain), while the human RNase 3 gene sequence was derived from a previously synthesized construct [67]. 3-[4,5-dimethylthiazol-2-yl]-2,5-diphenyl tetrazolium bromide (MTT), Isopropyl β-D-1-thiogalactopyranoside (IPTG), colistin and tobramycin were obtained from Apollo Scientific. Moxifloxacin was sourced from Acros Organics (Geel, Belgium). Mueller–Hinton broth (MHB) and resazurin were purchased from Merck (Kenilworth, NJ, USA). Crystal violet (CV) was obtained from Sigma-Aldrich (St. Louis, MO, USA). Calgary Biofilm Device (CBD) was acquired from Innovotech (Edmonton, AB, Canada).

### 4.2. Biofilm Formation

Biofilms were grown using the Calgary Biofilm Device (CBD) following the standard protocol [43], with minor adaptations. Briefly, an overnight culture of *A. baumannii* was diluted in MHB to an OD_600_ of 0.02 and 150 µL of the bacterial suspension was added to each well of a polypropylene 96-well plate. To minimize evaporation, the outer wells were filled with an equal volume of PBS. The CBD lid was then placed onto the plate, allowing the pegs to become immersed in the bacterial solution. The assembly was incubated at 37 °C with shaking at 100 rpm. A 24 h incubation period was routinely used to ensure the development of mature biofilms on the pegs.

### 4.3. Quantification of Biofilm Viable Cells by Resazurin-Based Assay

Biofilm viable cells were quantified using an optimized protocol of a resazurin-based method [35]. After growth or treatment, biofilms formed on CBD pegs were rinsed three times with PBS and then transferred to a new 96-well plate containing 200 µL of MHB supplemented with 16 µg/mL resazurin per well. Resazurin is irreversibly converted by bacterial oxidoreductases into the fluorescent metabolite resorufin and thus serves as an indicator of bacterial viability. The plate was incubated for 12 h in a Spark^®^ microplate reader (Tecan, Männedorf, Switzerland) at 37 °C with orbital shaking and fluorescence was measured every 10 min (excitation at 530 nm, emission at 590 nm).

For each condition, the time required to reach half of the maximum fluorescence signal was determined. To quantify the number of viable bacteria within biofilms, serial five-fold dilutions of an overnight bacterial culture were prepared in resazurin-containing MHB and added to a polypropylene 96-well plate for kinetic fluorescence measurement, also recorded every 10 min. These dilutions were plated on LB agar for bacterial quantification by colony counting. A correlation was then established between planktonic bacterial concentration and the time to reach half-maximal fluorescence. Finally, the viability of biofilm-embedded bacteria was estimated by converting the fluorescence-based half-max time into an equivalent number of planktonic cells as an approximation. Medium without antibiotic was used to subtract the background signal.

### 4.4. Antibiotic Challenge of Biofilms

Two-fold serial dilutions of the antibiotics (colistin, moxifloxacin and tobramycin) were prepared in 96-well plates using MHB, with a final volume of 200 µL per well. Mature biofilms formed on the CBD pegs were rinsed three times with PBS and then transferred to the antibiotic-containing plate for incubation at 37 °C with shaking at 100 rpm for 12 h. The range of concentrations tested were selected according to the planktonic MIC value, with starting values below the lethal dose of each antibiotic. Planktonic MIC values of each antibiotic were as follows; 1 μM for colistin, 8.51 μM for tobramycin and 0.31 μM for moxifloxacin. Following antibiotic exposure, biofilm viability was assessed using either the resazurin-based fluorescence assay or CV staining method.

### 4.5. Quantification of Biofilm Biomass by Crystal Violet (CV) Assay

Biofilm biomass was quantified by CV assay as previously described [68]. Briefly, following growth or antimicrobial treatment, biofilms formed on CBD pegs were rinsed three times with PBS, transferred to a dry 96-well plate and incubated at 60 °C for 1 h to fix the biomass. The biofilms were then stained with 200 µL of 0.1% CV for 15 min, rinsed three more times with PBS and dried by incubation at 37 °C for 30 min. CV was subsequently eluted with 200 µL of 33% acetic acid and the absorbance was measured at 595 nm using a Spark^®^ (Tecan) plate reader.

### 4.6. Recombinant RNase Expression and Purification

The pET11c plasmids containing RNase 3/1-v1 and RNase 3/1-v3 were taken from our previous work [32,34]. *E. coli* BL21 (DE3) strain was transformed with the plasmids for prokaryote high yield expression of the RNases. The recombinant proteins were expressed and purified as previously described [69], with some modifications [70]. The bacterial strain was grown in Terrific Broth (TB) medium containing 400 μg/mL ampicillin at 37 °C and constant agitation at 250 rpm. Once the culture reached an OD_600_ of 0.6, recombinant protein’s expression was induced for 4 h with 1 mM IPTG. Then, the pellet was collected and suspended in Tris–HCl 10 mM pH 8.5, 2 mM EDTA, left incubating for 30 min with 40 μg/mL of lysozyme, sonicated and centrifuged at 22,000× *g*. The resulting pellet was suspended in 25 mL of the same buffer with 1% Triton X-100 and 1 M urea and incubated for 30 min. After that, it was centrifuged at 22,000× *g* during 30 min at 4 °C and the supernatant was discarded. This process was repeated until the supernatant was clear. The Triton X-100 was removed by suspending the pellet in Tris–HCl 10 mM pH 8.5 and 2 mM EDTA, stirred during 30 min and centrifuging at 22,000× *g* for 30 min at 4 °C. The resulting pellet was suspended in 25 mL of Tris-HCl 100 mM, pH 8.5, 2 mM EDTA, 6 M guanidine hydrochloride and left overnight in constant agitation in order to solubilise the inclusions bodies. The day after, reduced L-glutathione (GSH) was added at 80 mM to the solution and incubated for two hours on a reducing nitrogen atmosphere. After incubation, the solution was centrifuged at 16,000× *g* during 30 min at 4 °C. The resulting supernatant was slowly added into 2 L of protein refolding buffer containing 100 mM Tris/HCl, pH 8.5, 0.5 M guanidinium chloride, L-Arginine-HCl 0.5 M and 2 mM oxidized glutathione (GSSG), having therefore a GSH/GSSG ratio of 4 and was left stirring during 48–72 h at 4 °C. The folded protein was buffer exchanged against 150 mM sodium acetate, pH 5 and purified by loading into a cationic exchange chromatography Resource S (GE Healthcare Life Sciences, Marlborough, MA, USA) column and eluting by a linear NaCl gradient from 0 to 2 M in 150 mM sodium acetate, pH 5. The purified RNases were then desalted, lyophilized and stored at 20 °C.

### 4.7. Determination of Bactericidal Activities of RNases Against Biofilms

Two-fold serial dilutions of recombinant RNases ranging from 200 to 0.01 µM were prepared in HBS, with a final volume of 200 µL per well in a polypropylene 96-well plate. Alternatively, a fixed concentration of 50 µM was used for all RNases. Biofilms grown on the CBD pegs were rinsed three times with PBS, transferred to the wells containing the RNase solutions and incubated at 37 °C with shaking at 100 rpm for 4 h. Following this bactericidal treatment, biofilm viability was assessed using the resazurin-based assay. To determine the concentration of planktonic bacteria released from the biofilms, 30 µL of the well supernatant was plated onto LB agar, incubated at 37 °C and colonies were subsequently counted.

### 4.8. Determination of RNase Activity as Colistin Adjuvants for Biofilms Eradication

Colistin was diluted in MHB to final concentrations ranging from 1 to 12 µM. Recombinant RNases were prepared at a concentration of 10 µM in a 0.01% acetic acid solution. Subsequently, 20 µL of the RNase solution was added to 180 µL of each colistin dilution, yielding a final RNase concentration of 1 µM. Biofilms grown on CBD pegs were rinsed three times with PBS, transferred to the wells containing the antimicrobial mixtures and incubated at 37 °C with shaking at 100 rpm for 12 h. Biofilm viability was then assessed using the resazurin-based assay.

### 4.9. Biofilm Resistance Evolution Assay by Colistin Exposition

Biofilms were grown in the CBD as previously described, starting from an overnight *A. baumannii* culture derived from a fresh isolated colony. A treatment plate was prepared by first adding 180 µL of MHB containing colistin, followed by 20 µL of a 10 µM RNase 3/1-v3 solution (to achieve a final concentration of 1 µM) suspended in 0.01% acetic acid, and incubated altogether. For negative controls, 180 µL of fresh MHB was added, and for RNase-free conditions, 20 µL of 0.01% acetic acid was included instead of RNase. Treated bacteria and negative controls were manipulated simultaneously cycle after cycle.

Biofilms grown on the CBD pegs were rinsed three times with PBS, transferred to the antimicrobial treatment plate and incubated at 37 °C with shaking at 100 rpm for 12 h. Following antimicrobial treatment, biofilm viability was monitored over the next 12 h using the resazurin-based assay in a Spark^®^ (Tecan) plate reader. Planktonic bacteria released from the biofilms during antimicrobial exposure were also assessed by measuring the OD_600_ of the treatment plate after each fluorescence cycle. The next day, biofilms were rinsed again three times with PBS, transferred to a fresh plate containing new antimicrobial treatments, incubated for an additional 12 h and subjected once more to resazurin fluorescence monitoring.

After fluorescence measurements, the bottom plate was stored at −80 °C with 15% glycerol for later analysis of evolving bacterial strains. The initial colistin concentration was set at 6 µM and was either maintained or increased during subsequent days based on biofilm viability: the dose was raised if no significant viability reduction was observed and kept constant if a substantial reduction occurred. Figure 6 outlines the main steps of the protocol.

### 4.10. Colistin MIC and MBEC Determination from Evolved Bacterial Lineages

Bacterial replicates stored throughout the antimicrobial resistance evolution assays were evaluated for colistin resistance acquisition by determining their MIC (minimum inhibitory concentration) and MBEC (minimum biofilm eradication concentration) values.

MIC was defined as the lowest antibiotic concentration that completely inhibited bacterial growth. Stationary-phase bacterial cultures were prepared by incubating the strains stored from days 3, 5, 7 and 9 of the biofilm resistance assay for 24 h at 37 °C with agitation at 100 rpm. Polypropylene 96-well plates were prepared with colistin dilutions in MHB ranging from 50 to 0.78 µM for low or non-resistant strains and from 1600 to 12.5 µM for highly resistant strains. Stationary cultures were diluted threefold and 2 µL of each dilution (approximately 2 × 10^7^ CFU/mL, resulting in a total 1:150 dilution) were inoculated into each well. Plates were incubated for 24 h at 37 °C with agitation and bacterial growth was assessed visually. Each strain’s MIC was determined in triplicate.

MBEC was defined as the lowest antibiotic concentration required to kill all bacteria within biofilms. Stationary-phase cultures were grown for 24 h at 37 °C with agitation at 100 rpm on the pegs of the CBD to allow biofilm formation. Only bacterial lineages stored from day 5 were tested for MBEC. Polypropylene 96-well plates containing colistin dilutions in MHB, ranging from 1597.44 to 1.56 µM, were prepared. Biofilms were rinsed three times with PBS before being transferred to the antibiotic-containing plates. After 24 h of incubation at 37 °C with shaking at 100 rpm, biofilms were rinsed again three times with PBS and transferred to plates containing fresh MHB supplemented with 16 µg/mL resazurin. Following an additional 24 h incubation, biofilm survival was assessed by visual inspection.

### 4.11. Statistics

Barlett’s test and Shapiro–Wilk’s method were used to study the variance homogeneity and normal distribution of the data, respectively. Linear regression was used to analyse correlations between bacterial counts and fluorescence signals. ED_50_ values were calculated by non-linear regression from dose–response curves. The Mann–Whitney U test was used to compare groups with non-parametric data, including MIC, MBEC and biofilm viability. Spearman’s correlation tested relationships between MIC, MBEC and biofilm biomass. The significance threshold was set at *p* < 0.05. All statistical analyses were performed using GraphPad Prism 8 (San Diego, CA, USA). All experiments were performed in at least triplicates.

## 5. Conclusions

Biofilm-based infections pose a significant challenge to public health, highlighting the demand for compounds capable of disrupting biofilm formation and preserving antibiotic efficacy. This challenge underscores the urgent need to develop robust experimental methods to evaluate novel drug candidates able to hinder resistance evolution in biofilm communities. In response to this requirement, we developed a biofilm resistance evolution assay that closely mimics the conditions of chronic infections, preserving the structural and physiological complexity of mature biofilms. The method optimized for *A. baumannii* enabled us to successfully assess the efficacy of an RNase chimera as a colistin adjuvant against resistance acquisition in biofilms. Notably, RNase 3/1-v3, combining high catalytic activity of human RNase 1 and high antimicrobial action of RNase 3, potentiated colistin anti-biofilm activity. Although the designed protein was previously demonstrated to inhibit the emergence of bacterial resistance in planktonic cultures, it could not fully prevent the development of colistin resistance in mature biofilms. Nonetheless, the protein promoted increased biofilm susceptibility to colistin and reduced the overall biofilm-forming capacity of the bacteria during the early stages of the resistance evolution assay. Overall, our findings emphasize the complexity of biofilm communities and outline the importance of developing novel methodologies to better understand resistance dynamics and accelerate the discovery of effective therapeutic strategies against biofilm-associated infections. The biofilm-resistance assay presented here should provide a novel valuable tool for evaluating the bacterial development of resistance to antimicrobial agents.

## Data Availability

The original contributions presented in this study are included in the article/Supplementary Materials. Further inquiries can be directed to the corresponding author(s).

## Supplementary Materials

The following supporting information can be downloaded at https://www.mdpi.com/article/10.3390/ijms27031512/s1.
