# Supplementary material for: A Novel Method to Monitor the Evolution of Antimicrobial Resistance in Acinetobacter baumannii Biofilms
Source: Int J Mol Sci. 2026 Feb 3;27(3):1512. doi: 10.3390/ijms27031512 (PMC12898484; doi:10.3390/ijms27031512)
Supplement: Supplementary file 1 [file ijms-27-01512-s001.zip › ijms-4089806-supplementary.pdf]

A Novel Method to Monitor the Evolution of Antimicrobial Resistance in *Acinetobacter baumannii* Biofilms

Raul Anguita, Jiarui Li, Ester Boix * and Guillem Prats-Ejarque *

Supplementary Materials:

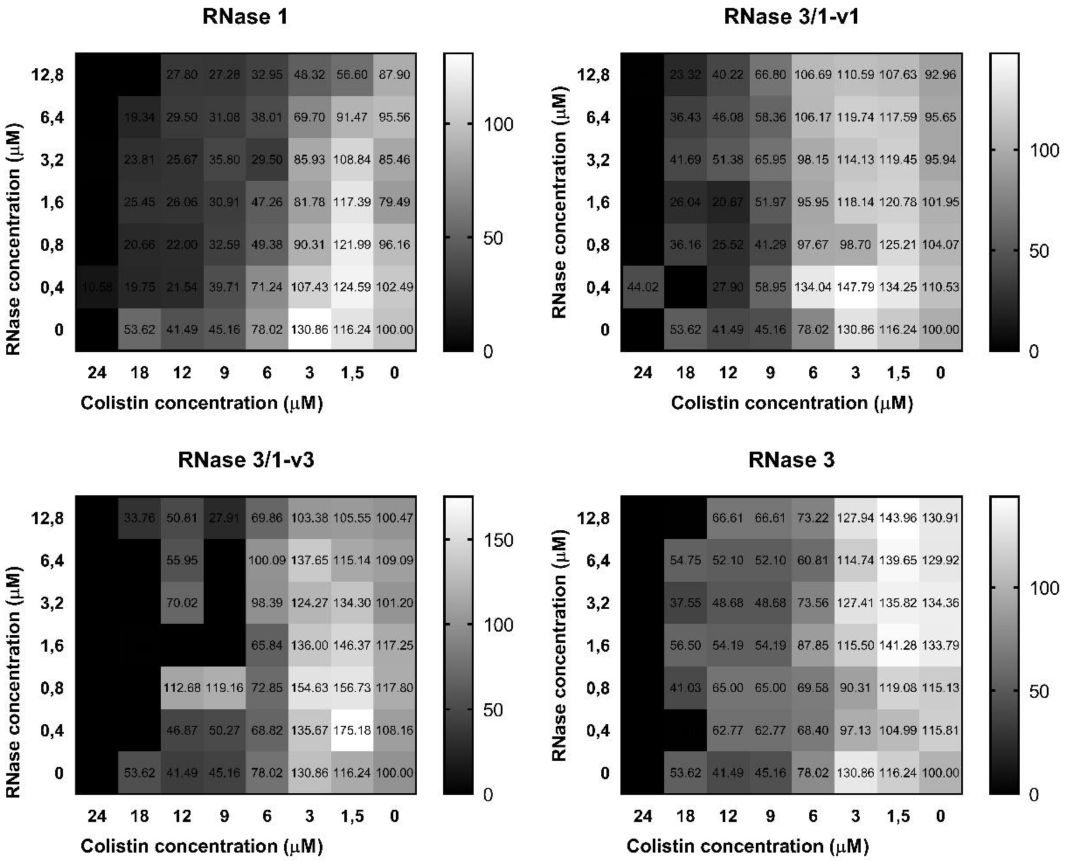

Figure S1. Combinatorial effects of parental and chimeric RNases on colistin-mediated biofilm eradication. Biofilms were treated with RNase concentrations ranging from 0.4 to 12.8 μM in combination with colistin concentrations ranging from 1.5 to 24 μM in MHB. Following treatment, biofilms were transferred to fresh MHB containing resazurin and viability was assessed via fluorescence monitoring. Results are presented as percentage viability relative to the untreated control.

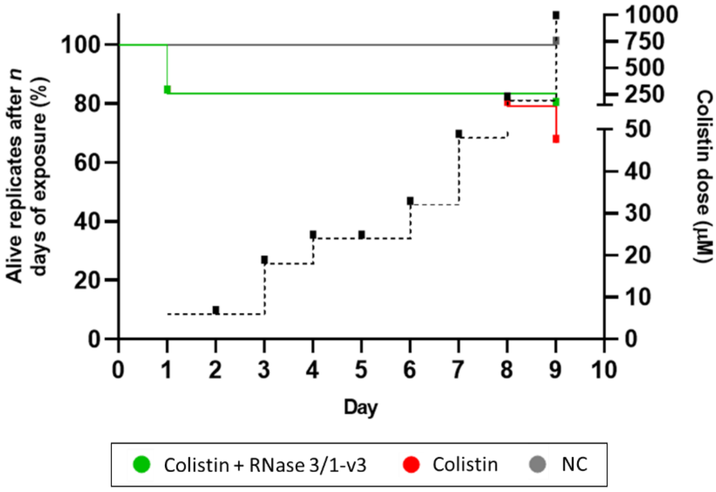

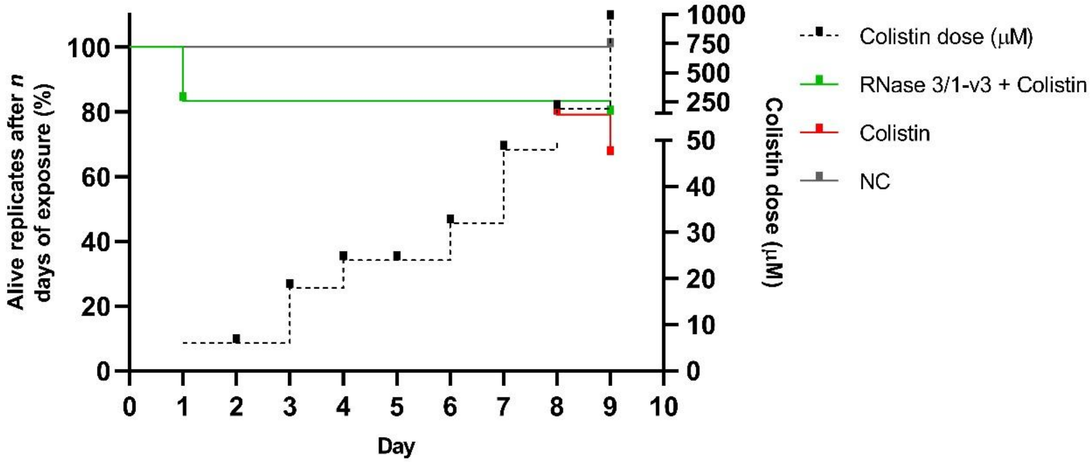

Figure S2. Graphical representation of surviving replicates after each day of exposure in the biofilm resistance evolution assay. The colistin dose used in each exposure is indicated in the right axis by the dotted line. RNase 3/1-v3, when used, was kept constant at 1 µM.

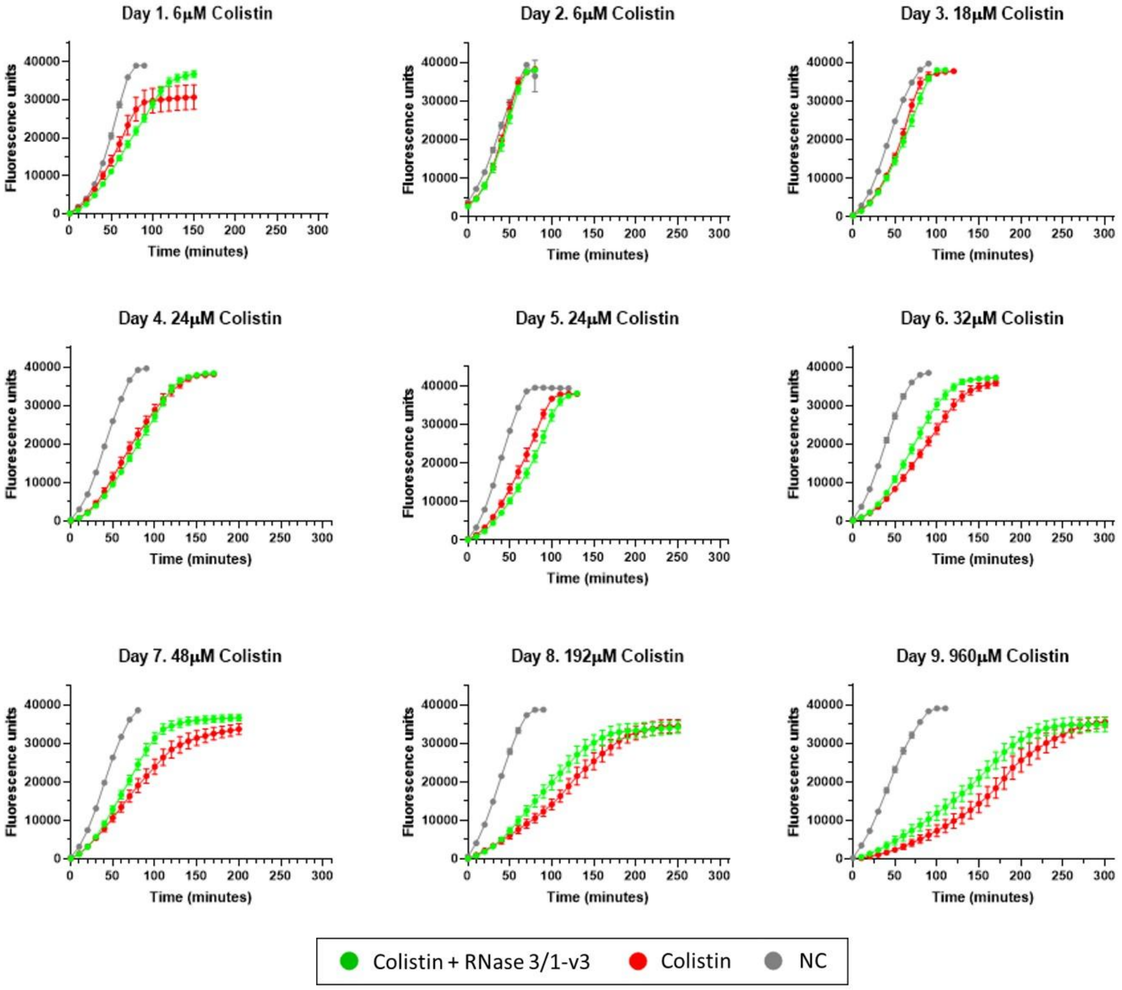

Figure S3. Resazurin fluorescence monitoring curves for each day of biofilm exposure to colistin, in presence or absence of RNase 3/1-v3. Biofilm viability was calculated for each condition and time point in the resistance evolution assay (Figure 4.7A), based on the time required to reach half of the maximum fluorescence signal. Values represent the recorded fluorescence mean of all the replicates. Error bars represent ± SEM.

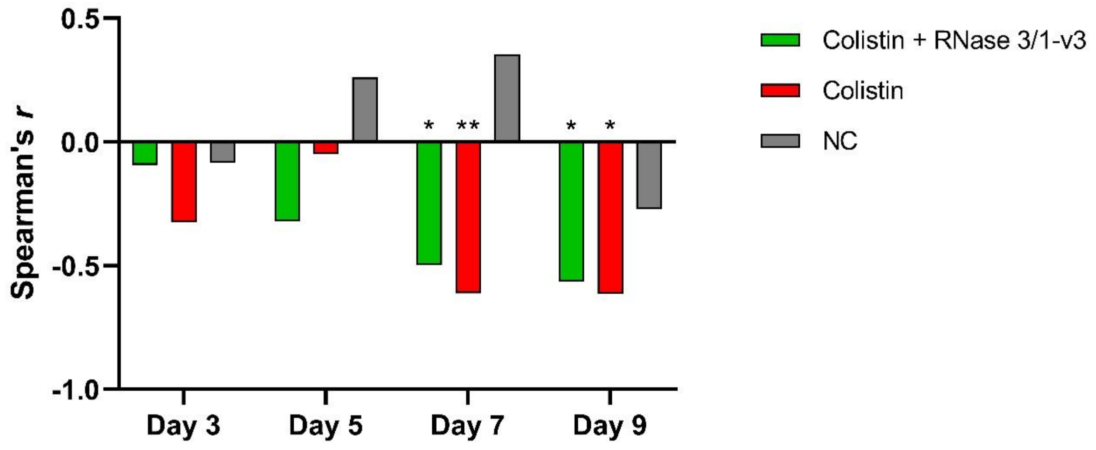

Figure S4. Correlation between biofilm-forming capacity and colistin resistance in planktonic bacteria derived from the biofilm resistance evolution assay. Biofilm biomass was quantified using CV assay and colistin resistance was assessed by MIC determination. Statistical correlations were calculated using the non-parametric Spearman's rank-order test and are indicated for each time point and data group.

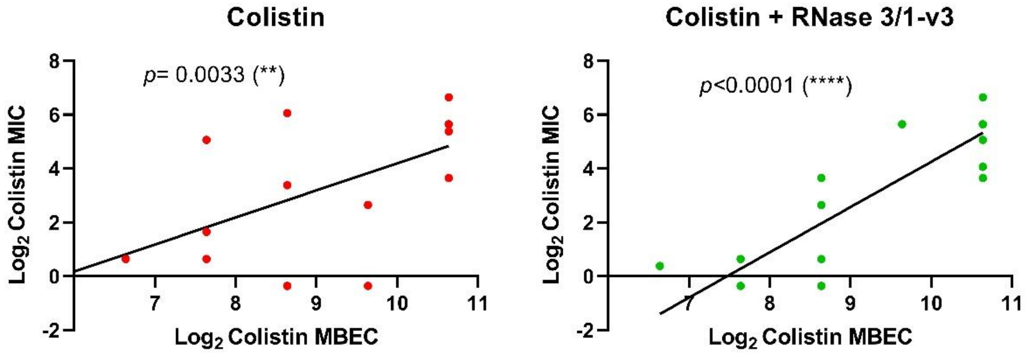

Figure S5. Correlation between MIC and MBEC values of bacterial lineages derived from day 5 of the biofilm resistance evolution assay. Statistical correlations were calculated using the non-parametric Spearman's rank-order test.
